# Pathological Changes in Uterine Fibroids

**Published:** 1909-07-24

**Authors:** 


### Gynæcology.

### PATHOLOGICAL CHANGES IN UTERINE FIBROIDS.

The importance of uterine fibroids as causes of serious illness and danger to life very often depends upon some degeneration occurring in the tumour. Much attention has recently been called to these changes, especially to necrobiosis, or red degeneration, and many operations have been recorded which have been rendered necessary on account of important symptoms set up by these changes. The hæmorrhage and pressure symptoms caused by fibroids in themselves may be serious enough to warrant radical operations, but they become doubly important if the patient suffers, in addition, from toxic symptoms fever and pain the result of degenerations. Of the changes more commonly seen in fibroids, necrobiosis has attracted most attention recently, although probably mucoid degeneration leading to cystic changes is more common. In addition to these, however, inflammatory changes (the result of infection), calcareous degeneration, and possibly sarcomatous degeneration, occur sufficiently often to deserve notice, especially with regard to the important symptoms they cause.

Necrobiosis, or red degeneration, shows itself as a real death of the fibro-muscular tissue, leading to loss of staining power and gradual disintegration. It was formerly supposed to be aseptic and dependent upon some change in the blood supply of the tumour whereby the growth was deprived of circulating blood, and consequently underwent necrosis. Recently, however, it has been suggested that the primary change is due to thrombosis of the veins in the capsule of the tumour, possibly the result of sepsis, and various organisms have been described as being the cause of this. The reports of these cases, however, are not beyond criticism, and at present it cannot be accepted without reserve that red degeneration has a microbial origin. There is no doubt, however, that the co-existence of pregnancy and fibroids is responsible for a large number of instances of this change, and the diversion of the blood-flow from the tumour to the pregnant uterus seems to be a natural explanation of the phenomenon. The uterine contractions occurring throughout pregnancy must exert some influence upon the nutrition of fibroids, and the constant associated anæmia in the tumour finally reduces its nutrition to the lowest ebb.

The appearance of a necrobiotic fibroid is characteristically red, contrasting very clearly with the normal greyish-white colour. This is especially the case when the change is only partial, as often is the case. When cut into fresh, these red areas are said to give out a "fishy" odour, quite unlike the smell of decomposition in gangrenous fibroids. Microscopically, the change is essentially a necrosis without any liquefaction of the elements of the tumour. The individual muscle-cells and connective tissue fibres can still be recognised, but they stain only with counterstains like eosin, and their nuclei have quite lost their staining power with basic dyes like hæmatoxylin.

The symptoms produced by necrobiosis are characteristic and are primarily two—namely, pain and rises of temperature. As fibroids, apart from the pressure they may cause, are usually painless and unaccompanied by fever, these two symptoms are sufficiently striking to draw attention to the change in the tumour, and are often important indications for radical operations. The pain must be ascribed to tension in the capsule of the tumour due to swelling of its contents, and in some cases at least this is contributed to by thrombosis of vessels and subsequent effusion of plasma in between the tumour and its capsule. The fever is more difficult to explain, unless the septic theory of the change is eventually proved to be correct. Apart from this, it is reasonable to expect that there must be absorption of products from the necrosing tissues, even if aseptic, just as absorption of effused blood will sometimes cause rises of temperature.

The treatment of necrobiotic fibroids has hitherto been removal, either with or without the uterus, and the results of the operation are exceedingly good. There is no evidence to show what would happen to these tumours if left alone, and how long the change can be permitted to go on before the patient becomes seriously ill. It is possible that a necrobiotic fibroid may be gradually absorbed, and this may be the explanation of the supposed disappearance of fibroid tumours, especially as it has been believed that these tumours sometimes disappear after pregnancy and labour. Given a case, however, in which these changes were suspected, we should hardly be justified in condemning a patient to prolonged invalidism in the hope that the tumour would eventually be absorbed. If necrobiosis occurs, then in all cases the tumour should be removed by operation; and from all published records as good a result may be expected as if the tumour were not in a degenerate condition.

Mucoid degeneration is the change in a fibroid which produces cystic cavities, such as used to be called fibro-cystic tumour of the uterus. It is not especially connected with pregnancy in the same uniform way as necrobiosis. Nothing definite is known as to the cause of mucoid degeneration, but it is usually ascribed to deficient blood supply to the tumour. This is quite incapable of proof, for it is not found only in pedunculated fibroids where partial strangulation might be expected, but quite as commonly in interstitial tumours. Macroscopically it produces rapid enlargement of the tumour and cavities containing yellowish, clear fluid. These cavities, at first small, run together and may produce large cysts, with ragged walls and often with strands of unaltered tissues running across them. These cysts are not the dilatation of pre-existing cavities, because they never have epithelial linings. Microscopically the change is seen to occur in the fibrous tissue first. The fibrils lose their character and become hyaline, whilst cells and nuclei disintegrate and form fluid droplets. Meanwhile the muscle fibres, deprived of nutritive fluids, likewise disintegrate and add to the accumulation. The tissue thus changing never assumes the red colour above described, but has a dirty yellowish-white appearance, often semi-translucent, and breaking down easily under pressure of the finger. The production of cysts often complicates the diagnosis of these cases, for the tumour may be so large and may fluctuate so freely as to be indistinguishable from an ovarian cyst. Apart from the rapid enlargement and consequent pressure on neighbouring parts, there are no important symptoms associated with this mucoid change. The menorrhagia of fibroids continues just the same when mucoid degeneration occurs, if the tumour is submucous or interstitial. The only treatment to be recommended is complete removal of the tumour and uterus if necessary.

